# Adipose-derived mesenchymal stem cells from the sand rat: transforming growth factor beta and 3D co-culture with human disc cells stimulate proteoglycan and collagen type I rich extracellular matrix

**DOI:** 10.1186/ar2473

**Published:** 2008-08-11

**Authors:** Hazel Tapp, Ray Deepe, Jane A Ingram, Marshall Kuremsky, Edward N Hanley, Helen E Gruber

**Affiliations:** 1 Department of Orthopaedic Surgery, 1000 Blythe Blvd, Carolinas Medical Center, Charlotte, NC 28232, USA

## Abstract

**Introduction:**

Adult mesenchymal stem cell therapy has a potential application in the biological treatment of disc degeneration. Our objectives were: to direct adipose-derived mesenchymal stem cells (AD-MSC) from the sand rat to produce a proteoglycan and collagen type I extracellular matrix (ECM) rich in known ECM components of the annulus fibrosis of disc; and to stimulate proteoglycan production by co-culture of human annulus cells with AD-MSC.

**Methods:**

AD-MSC were isolated and characterised by adherence to plastic, appropriate expression of cluster of differentiation (CD) markers, and differentiation to osteoblasts and chondrocytes *in vitro*. AD-MSC were grown in three-dimensional (3D) culture and treated with or without transforming growth factor beta (TGFβ) to direct them to produce annulus-like ECM as determined by proteoglycan content and collagen expression. AD-MSC were co-cultured with human annulus cells and grown in 3D culture.

**Results:**

AD-MSC produced a proteoglycan and collagen type I rich ECM after treatment with TGFβ in 3D culture as confirmed by a 48% increase in proteoglycan content assayed by 1,9-dimethylmethylene blue (DMB), and by immunohistochemical identification of ECM components. Co-culture of human annulus and sand rat AD-MSC in 3D culture resulted in a 20% increase in proteoglycan production compared with the predicted value of the sum of the individual cultures.

**Conclusion:**

Results support the hypothesis that AD-MSC have potential in cell-based therapy for disc degeneration.

## Introduction

Previous research has shown that adult mesenchymal stem cells have the potential for biological cell-based treatment of disc degeneration [1,2]. Degenerated discs have a decreased proteoglycan content associated with a loss of load-bearing function. Harvesting disc cells from the acellular disc tissue is difficult because of the low numbers of disc cells and many of the cells show senescence [3], programmed cell death [4], or decreased or altered extracellular matrix (ECM) expression [5].

Stem cells are characterised by their ability to differentiate into lineage-specific cell types [6-8]. Bone-marrow derived mesenchymal stem cells (BM-MSC) transplanted to degenerative discs in rabbits were found to proliferate and differentiate into cells expressing some of the major extracellular components of discs [9]. BM-MSC injected into canine discs was partially effective in inhibiting disc degeneration and may be responsible for maintaining disc immune privilege [10].

Adipose-derived mesenchymal stem cells (AD-MSC) offer some advantages as an attractive, readily available adult stem cell because of the ease of harvest and their abundance [11,12]. AD-MSC are capable of differentiating into adipocytes, chondrocytes and osteoblasts, and, more recently, have been shown to differentiate into insulin-, somatostatin- and glucagon-expressing cells [13]. AD-MSC have great potential as a carrier for therapeutic growth factors. For example, AD-MSC genetically modified by bone morphogenic protein 2 (BMP-2) produced a significant increase of newly formed bone in a canine bone defect study [14]. Some of the most potent inducers of chondrogenic differentiation are members of the transforming growth factor-beta (TGFβ) super family such as the TGFβ isoforms and the BMPs [15]. Also important are the fibroblast growth factor isoforms and insulin-like growth factor (IGF-1).

TGFβ super family cytokines act through binding to cell-surface receptors. Differentiation occurs through two major intracellular pathways: through the small mothers against decapentaplegic homolog (SMAD) signalling transcription factors; and through mitogen activated protein kinase. Synergistic interactions between TGFβ and other cytokines, such as IGF1, has been reported [16]. The IGF-1-activated signalling cascade is hypothesised to interact with the TGFβ pathway. Although the precise mechanism of action of TGFβ has not been elucidated, the key events responsible for the differentiation of mesenchymal cells to the chondrogenic lineage are known to take place during the first days of growth factor exposure [17]. TGFβ1 is a standard media additive used in culture to induce chondrogenesis. TGFβ3 has been shown to induce a more rapid and representative expression of chondrogenic markers [18].

Little is known about the effect of stem cells, or stem cell-conditioned media (CM), on disc cells. Previous experiments with disc cells have shown that co-culture of nucleus pulposus with annulus fibrosis cells stimulated proliferation. Reinsertion into the discs of rabbits retarded disc degeneration [19]. Other work has demonstrated an increased synthesis of proteoglycans after pellet co-culture of disc cells with BM-MSC [20]. Stimulation of disc cells with stem cells or CM could enhance the success of autologous implantation of disc cells.

In the present study we use two approaches to investigate disc remediation via disc or stem cell stimulation. First, we extract, characterize and stimulate AD-MSC obtained from the sand rat with TGFβ treatment in 3D collagen sponges to produce a proteoglycan and collagen type I ECM, rich in known disc ECM components. Second, we investigate the matrix stimulatory effect of AD-MSC co-cultured in 3D culture with human annulus fibrosis cells.

## Materials and methods

### Source of fat tissue

Animal studies were performed following approval by the Carolinas Medical Center Institutional Animal Care and Use Committee. *Psammomys obesus*, the sand rat, is used in our laboratory in studies of disc degeneration. Colony housing and animal diet descriptions have been published previously [21,22]. Immediately after euthanasia, adipose tissue from the back and inguinal areas was surgically obtained using sterile techniques, placed in a petri dish containing Hank's Buffered Salt Solution ([HBSS] Gibco, Carlsbad, CA) and rapidly transported to the laboratory. Approximately 2 g of fat tissue was obtained per harvest and processed as described below.

### AD-MSC isolation and plating

Cell culture methods were adapted from the method described by Cowan et al. [2]. Fat was placed in a sterile petri dish, minced well in HBSS, and digested with 1 mg/ml collagenase type II (Sigma, St. Louis, MO) at 37°C in a water-bath shaker for 30 to 40 minutes at 180 to 200 rpm with a brief vortex every 10 minutes. Undigested tissue was removed by filtering through 100 μm nylon cell strainers (Falcon, Franklin Lakes, NJ). Multipotent AD-MSC were harvested by centrifugation at 42 *g *for five minutes at room temperature. The pellet was then resuspended in 2 ml HBSS, filtered through a 40 μm cell strainer, counted and plated as the primary culture (P0) on 100 × 20 mm round plastic tissue culture dishes (Primera, Falcon, BD Biosciences, San Jose, CA) at a density of 1000 cells/mm^2^. A density of 1000 cells/mm^2 ^was chosen as a high enough density for cell to cell contact but low enough to allow space for several days of proliferation without the cells becoming confluent. Cells were fed every 48 to 72 hours with 10 ml media (Mesemchymal Stem Cell Basal Media [MSCBM], Cambrex Bio Science, Walkersville, Baltimore, MD). When confluent, cells were trypsinised, centrifuged at 42 *g *for five minutes and re-plated at a density of 1000 cells/mm^2^.

### Verifying stem cell isolation

#### CD marker analysis of AD-MSC

AD-MSC were characterised by localisation of the multipotent mesenchymal stem cell markers CD105 and CD29 and negative localisation of CD45 and CD34 [23]. For cluster of differentiation (CD) immunohistochemical assessment of stem cell markers, AD-MSC were grown on two- and four-well Nunc slides (Nalge Nunc international, Rochester, NY) (Table 1). AD-MSC were harvested for CD analysis by scraping them off the surface with plastic pipette tips. They then underwent centrifugation at 42 *g *for five minutes, resuspension in 1% agarose (Sigma, St. Louis, MO), fixation with 10% neutral buffered saline (Allegiance, McGaw Park, IL) for 20 minutes, followed by storage in 70% ethanol (AAPER, Shelbyville, KY) until processed for paraffin embedding.

**Table 1:** Profile of antibodies used in 3D immunohistological and CD marker studies

**Antibody**	**Source**	**Dilution**
Type I collagen	Biodesign International (Kennebunk, ME)	20 μg/ml
Type II collagen	Biodesign International (Kennebunk, ME)	20 μg/ml
Decorin	R&D Systems, (Minneapolis, MN)	25 μg/ml
Keratin sulphate	Seikagaku Corporation, (Tokyo, Japan)	5 μg/ml
Chondroitin sulphate	ICN Biomedicals, (Costa Mesa, CA)	20 μg/ml
CD29	Lab Vision Corporation (Fremont, CA)	200 μg/ml
CD34	DakoCytomation (Carpinteria, CA)	50 μg/ml
CD45	DakoCytomation, (Carpinteria, CA)	350 μg/ml
CD105	Lab Vision Corporation (Fremont, CA)	200 μg/ml

3D, three dimensional; CD, cluster of differentiation.

The CD antibodies work to identify cells by immunohistochemical visualisation. CD surface marker identification, along with plastic adherence and lineage specific differentiation satisfy the standard criteria suggested for defining mesenchymal stem cells. Mesenchymal stem cells should characteristically show positive localisation of CD44, CD29, CD105 and CD90, but no localisation of haematopoietic markers CD45, CD34 [23]. These sand rat-derived AD-MSC are positive for the markers CD29 and CD105 and negative for the haematopoietic markers CD45 and CD34. The readily available anti-human markers CD90 and CD44 did not cross-react with sand rat tissue; thus they were not tested in the present study. Since anti-sand rat antibodies for CD markers are not commercially available, the CD markers listed in Table 1 were first tested for use by appropriate reactivity against sand rat lymph nodes.

In order to further verify the lineage plasticity of AD-MSC, osteogenic and chondrogenic differentiation was confirmed using standard methods described below.

#### Osteogenic differentiation

Osteogenic differentiation of stem cells using an osteogenesis kit (Chemicon International, Temecula, CA) [24] was confirmed by positive alizarin red staining of mineralised matrix after 21 days of culture. Control cultures were only fed MSCBM media.

#### Chondrogenic differentiation using micromass culture

Cells were grown for seven to 10 days in chondrogenic induction medium (Cambrex Bio Science, Walkersville, Baltimore, MD) supplemented with 5% fetal calf serum (FCS). They were harvested for histological examination, embedded in agarose, pellets fixed with 10% neutral buffered saline for 20 minutes and stored in 70% ethanol until processed for paraffin embedding. Proteoglycan production in the ECM was visualised by toluidine blue staining (Sigma, St. Louis, MO; 0.1% in distilled water).

### Stimulation of AD-MSC to increase proteoglycan and collagen type I production

To increase proteoglycan and collagen type I production, 3D cell culture and exposure to TGFβ were used.

#### Growth and differentiation of stem cells in 3D scaffold culture

Sterile collagen sponge (Gelfoam, Pharmacia & Upjohn Co, Kalamazoo, MI, USA), an absorbable collagen sponge prepared from purified pig skins previously used in our laboratory to grow intervertebral disc cells in 3D culture [25], was used as a 3D scaffold. AD-MSC were suspended in MSCBM at a concentration of 1 × 10^7 ^cells/ml. Droplets of 10 μl (containing 1 × 10^5 ^cells) were injected into collagen sponges trimmed into 0.5 cm^3 ^cubes. An optimum number for maximum proteoglycan production in collagen sponge has previously been found to be 1 × 10^5 ^cells/0.5 cm^3 ^of collagen sponge [25]. Replicate collagen sponges were placed on Costar Transwell Clear Inserts (Corning Incorporated-Life Sciences, Lowell, MA) in 24-well plates and fed three times per week with 2.0 ml of MSCBM with 10 ng/ml TGFβ (Cambrex Bio Science, Walkersville, MD) or without TGFβ (control). The typical dose of TGFβ used in the literature for chondrogenic differentiation is 10 ng/ml [17]. Cells were grown for two to six weeks and assayed for proteoglycan production in the presence or absence of TGFβ. Cultures were terminated, fixed in 10% neutral buffered saline for one hour and embedded in paraffin. Collagen sponge was sectioned for immunohistochemical analysis and stained for ECM proteoglycan production using toluidine blue (Sigma, St. Louis, MO; 0.1% in distilled water). Proteoglycan production was also assessed using the 1,9-dimethylmethylene blue (DMB) assay and by scoring ECM production after immunohistochemistry.

Cell proliferation was evaluated by seeding AD-MSC in a monolayer in 48-well tissue culture plates at known cell densities and treating with MSCBM in the presence or absence of TGFβ. After five days of culture, wells were rinsed and held at -80°C. A FluoReporter Blue Fluorometric dsDNA Quantitation kit (Molecular Probes Inc, Eugene, OR) was used to assess cell proliferation per manufacturer's directions. Tests were run in duplicate for each culture and results averaged for statistical analysis.

#### Assay of total sulphated glycosaminoglycan production

Cells were grown in 3D culture for 14 days in the presence or absence of TGFβ and assayed for sulphated proteoglycan production using the DMB assay [26].

#### Scoring of immunohistochemistry and toluidine blue staining

Scoring of slides from immunohistochemical staining of cell-surface markers, ECM proteins and toluidine blue staining of total proteoglycans was performed blinded by HG, HT and MK. The following scoring scale was used: 1 = very slight localisation; 2 = modest localisation; 3 = abundant localisation. For accuracy and consistency, previously scored examples of grades were reviewed before each scoring session; in addition, random previously scored slides were re-scored to assure consistency.

### Immunohistochemistry

Specimens were fixed in 10% neutral buffered saline for one hour, transferred to 70% ethyl alcohol and held for paraffin processing using a Shandon Pathcentre Automated Tissue Processor (ThermoShandon, Pittsburgh, PA). Collagen sponges were bisected and the two halves embedded on edge. Specimens were embedded in Paraplast Plus paraffin (ThermoShandon, Pittsburgh, PA), and 4 mm serial sections cut with a Leica RM2025 microtome (Nussloch, Germany) and mounted on Superfrost-Plus microscope slides (Allegiance, McGaw Park, IL).

Immunohistochemical localisation of CD markers, types I and II collagen, chondroitin sulphate, decorin and keratin sulphate utilised antibodies used techniques described previously [27] (Table 1). Negative controls consisted of rabbit IgG (Dako, Carpinteria, CA; for collagen I and II) or mouse IgG (Dako, Carpinteria, CA; for all other antibodies) used at the same concentration as each tested antibody.

### 3D co-culture of AD-MSC and human disc cells

Human disc cell studies were performed following approval by the Carolinas Medical Center's human subjects Institutional Review Board (IRB Protocol # 08-04-09E). The need for informed consent was waived because surgical tissue is routinely discarded at our institution.

To assess the effect of AD-MSC on human annulus disc cells, a 3D co-culture system was used to measure ECM and proteoglycan changes when disc cells were co-cultured in contact with AD-MSC or grown in CM previously used to feed monolayer AD-MSC cultures. Human annulus cells from surgically removed lumbar disc tissue (Thompson grades 3 or 4 [28]) were obtained from four surgeries and established in culture as previously described [29]. Flasks of confluent annulus cells were rinsed twice with phosphate buffered saline and labeled *in situ* with carboxyfluorescein diacetate succinimidyl ester (CFSE) (10 μM for 10 minutes at 37°C) using established methods [5,22,30].

Replicate samples of resuspended AD-MSC, labelled annulus cells, or premixed AD-MSC and annulus cells were injected into collagen sponges as described above. Cultures and co-cultures were soaked with 2.0 ml of MSCBM, CM or a 50:50 mixture of the two, and were fed three times per week for two weeks. Cultures were then assayed for proteoglycan production by DMB assay. To calculate proteoglycan production in co-culture, data was expressed as an increase in sulphated proteoglycans compared with the predicted value taken as the sum of the individual control stem and disc cultures [31].

### Statistical analysis

Data were analysed using SAS version 8.2 (SAS, USA). A p < 0.05 was considered statistically significant. Standard statistical methods were used. Data are presented as mean ± SD (n).

## Results

### Morphology of AD-MSC in monolayer culture

AD-MSC were plated and observed 24 hours a day for three days and at one week after tissue extraction (data not shown). After attachment, the cells gradually spread out and assumed the fibroblastic morphology previously reported for stem cells [32]. Cells became confluent after approximately one week. With sequential passaging, the rate of cell proliferation gradually slowed. The time between passages lengthened from seven days for P1 and P2, 10 days for P3 to three weeks for P4 to P6. The total number of passages before cell growth diminished varied according to the age of the donor sand rat. In general, AD-MSC from younger sand rats up to age 12 months were passaged six to eight times; AD-MSC from sand rats older than 12 months were usually only passaged up to three times.

### Characterisation of stem cells

AD-MSC were characterised using the following accepted criteria [23]: adherence to plastic; osteogenic and chondrogenic differentiation as evidence of multipotent differentiation potential; and specific surface antigen expression.

***Adherence to plastic ***Cells attached well and displayed a fibroblastic-like morphology in monolayer culture by three days.

**Figure 1:**
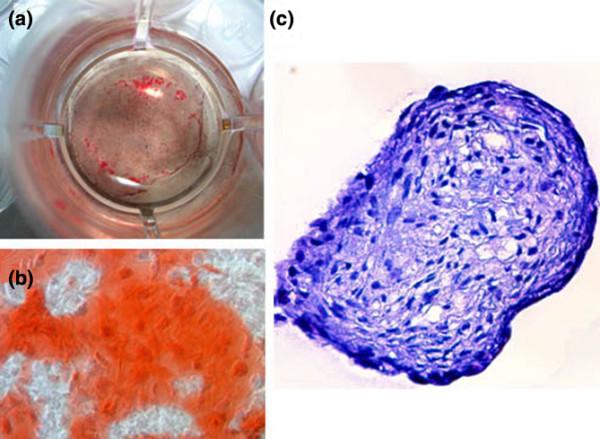
Osteogenic differentiation of adipose-derived mesenchymal stem cells (AD-MSC). **(a) **and **(b) **AD-MSC treated with osteogenic media for three weeks stained with Alizarin red. Red staining marks mineralised matrix produced by osteoblasts. A at × 4 magnification; (b) at × 105 magnification. **(c) **High density cultures showed formation of a chondrogenic phenotype when cultured in micromass; pink extracellular matrix staining marks proteoglycans stained with toluidine blue × 95.

***Osteogenic differentiation ***of AD-MSC was confirmed by alizarin red staining of mineralised bone matrix deposited *in vitro* after feeding AD-MSC with commercially available osteogenic inducing media (Figures 1a and 1b).

***Chondrogenic differentiation ***of AD-MSC was confirmed by formation of chondrogenic micromasses in culture and by positive proteoglycan staining of ECM produced by cells within the micromasses (Figure 1c). In addition, monolayer AD-MSC grown in chondrogenic media showing a more rounded phenotype typical of chondrocytes (data not shown).

**Figure 2:**
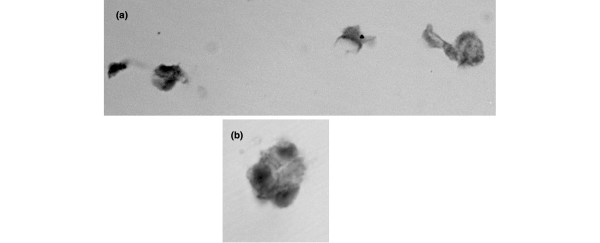
Immunolocalisation of the mesenchymal stem cell markers from passage one adipose-derived mesenchymal stem cells grown in monolayer. **(a) **CD29 at × 650; **(b) **CD105 at × 840.

***CD marker analysis ***AD-MSC were also characterised by localisation of the multipotent mesenchymal stem cell markers CD105 (approximately 75% of cells were positive) and CD29 (more than 90% of cells were positive) (Figure 2) and negative localisation of CD 45 and CD34 (data not shown).

### Stimulation of AD-MSC by TGFβ

After being established in monolayer, AD-MSC were stimulated to produce a proteoglycan rich ECM by 3D culture in a collagen sponge and treatment in the presence or absence of TGFβ.

#### Cell morphology in monolayer

**Figure 3:**
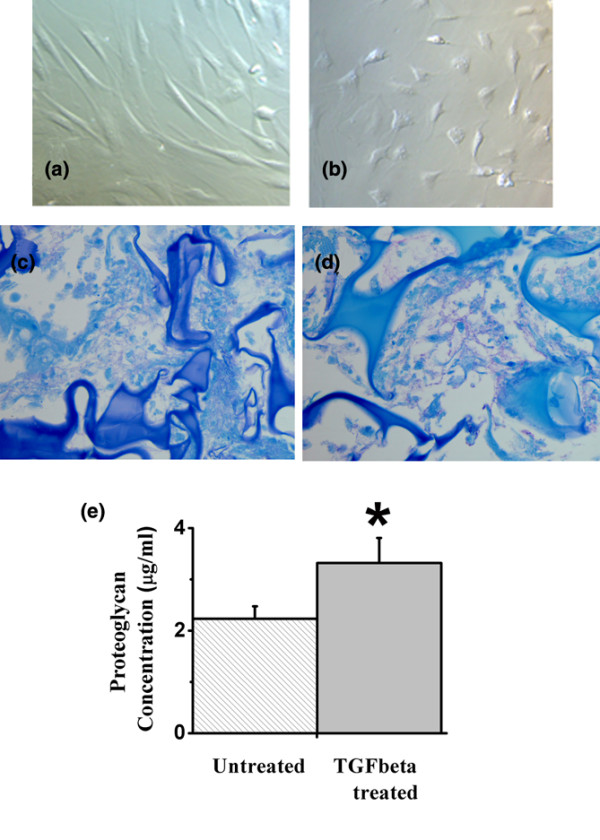
Effect of transforming growth factor beta (TGFβ) in culture. **(a) **Monolayer culture. Cells show typical fibroblast-like morphology in control culture × 180. **(b) **Monolayer culture. In the presence of TGFβ, morphology changes to a more rounded phenotype × 155. **(c) **3D culture. In the absence of TGFβ, extracellular matrix contains modest amounts of proteoglycans (indicated by pink staining with toluidine blue) × 400. **(d) **3D culture. Cells produce abundant proteoglycan when cultured with TGFβ × 400. **(e) **Quantitative analysis of proteoglycan shows significantly greater formation in the presence of TGFβ. * p < 0.05.

Within one week of monolayer culture, TGFβ-treated AD-MSC became rounded and less fibroblast-like in appearance (Figures 3a and 3b). Compared with control AD-MSC, where confluence was observed in two to three weeks, proliferation of AD-MSC in the presence of TGFβ slowed gradually, and cells did not achieve confluency (data not shown).

#### Cell morphology in 3D culture

When grown in 3D culture, morphological studies of AD-MSC showed that the AD-MSC grew as rounded cells filling the cavities in and around the 3D matrix (unlike the typical fibroblastic morphology of monolayer-cultured AD-MSC). Cells cultured within the 3D sponge were rounded, whereas cells attached to the outer sponge margins were more flattened and elongated (Figures 3c and 3d).

#### Biochemical measurement of proteoglycan production and proliferation: effect of 3D growth and TGFβ

Evaluation with the DMB assay showed that TGFβ-treated AD-MSC in 3D culture had 48% more proteoglycan compared with AD-MSC in 3D culture alone (p < 0.05; Figure 3e). The FluoReporter quantitation assay of cell proliferation showed a four-fold increase in the number of AD-MSC after four days in monolayer culture (data not shown) for both TGFβ treated and untreated AD-MSC with no further increase after seven days. No significant difference in proliferation was seen for TGFβ-treated AD-MSC compared with untreated AD-MSC.

#### Morphological studies: effect of TGFβ on AD-MSC proteoglycan production

Toluidine blue staining showed the presence of ECM proteoglycan between and around the AD-MSC (Figures 3c and 3d). Extensive ECM was present at the edges of the 3D collagen sponge; ECM grading showed that the quantity of ECM increased between two and four weeks in culture. TGFβ treatment also increased levels of ECM in 3D culture. Semi-quantitative grading of toluidine blue-stained AD-MSC in 3D cultures showed the TGFβ-treated AD-MSC scored significantly higher (2.9 +/- 0.15), than the control slides (1.63 +/- 0.20) cultured without TGFβ (Figures 3c and 3d; Table 2). Paired *t*-test analysis showed a significant increase in ECM production for the TGFβ-treated samples (p < 0.05). Sand rat age did not correlate with ECM proteoglycan levels for either TGFβ-treated or control AD-MSC (Table 2).

**Table 2:** Histological grading of proteoglycan extracellular matrix formation by adipose-derived mesenchymal stem cells (AD-MSC) seeded into 3D matrix^a^

**Age of donor sand rat**	**Number of weeks of cultured**	**Mean histological grading score (mean +/- standard deviation)**	
		
		**- TGFβ**	**+ TGFβ**^b^
10 weeks	3 to 5	2.0 ± 0.0 (3)	3.0 ± 0.0 (3)
6 months	3 to 6	1.0 ± 0.0 (3)	2.7 ± 0.47 (3)
20 months	3 to 4	1.3 (2)	3.3 (2)

^a^Scoring scale: 0 = no localization; 1 = very slight localization; 2 = modest localization; 3 = abundant localisation. ^b^Transforming growth factor beta (TGFβ) treated AD-MSC gave a significantly higher score compared with untreated cells (p < 0.05).

#### Effect of TGFβ on AD-MSC extracellular matrix (immunohistochemical analysis)

**Figure 4:**
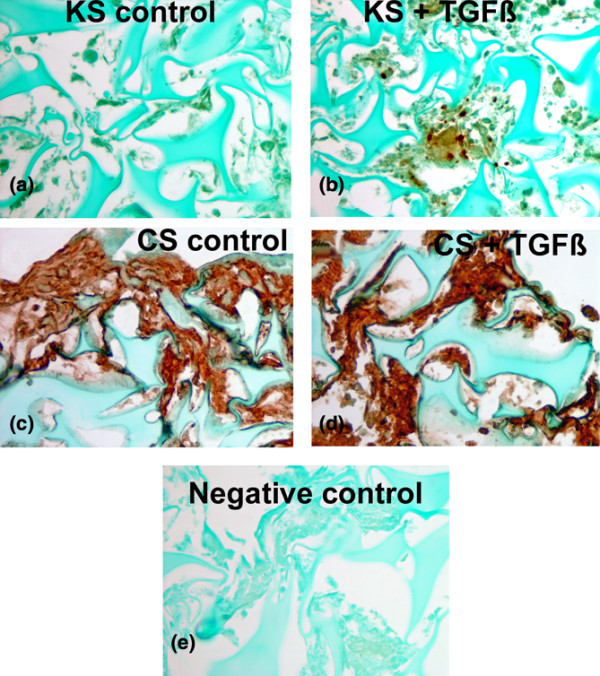
Immunohistochemical documentation of extracellular matrix formed by sand rat adipose-derived mesenchymal stem cells in 3D culture in the presence **(b, d) **or absence **(a, c) **of transforming growth factor beta (TGFβ). Note the enhanced keratin sulphate (KS) formed when TGFβ is present (b). Chondroitin sulphate (CS) also was enhanced with TGFβ (d) compared with control (c) × 360. Immunolocalization product is brown.

Immunohistochemical evaluation confirmed the presence of chondroitin sulphate and keratin sulphate (Figures 4a–d), types I and II collagen and decorin (data not shown). Localisation of the ECM proteins was present in cell layers on margins of the 3D matrix and between cells within the 3D matrix (Figure 4). More intense localisation for all ECM proteins was associated with TGFβ-treated 3D matrix samples. Slide scoring showed greater ECM for TGFβ-treated 3D matrix samples (Table 3). Paired *t*-test analysis from four experiments showed TGFβ-treated 3D cultured samples had significantly higher scores for collagen I, keratin sulphate and decorin (but not for chondroitin sulphate and collagen II) (Table 3; p < 0.05).

**Table 3:** Immunohistochemical characterisation of extracellular matrix (ECM) formed by adipose-derived mesenchymal stem cells (AD-MSC) in 3D culture for two to five weeks^a^

**ECM protein**	**Mean histological grading score** **Mean of four experiments +/- standard deviation**	
	
	**- TGFβ**	**+ TGFβ**
Keratin sulphate^b^	1.5 ± 0.58	2.5 ± 0.58
Chondroitin sulphate	2.5 ± 0.58	3.0 ± 0.00
Collagen^b ^type I	2.4 ± 0.42	3.0 ± 0.00
Collagen type II	2.4 ± 0.42	2.6 ± 0.42
Decorin^b^	1.5 ± 0.58	2.3 ± 0.50

^a^Scoring scale: 1 = very slight localization; 2 = modest localisation; 3 = abundant localisation. ^b^Tranforming growth factor beta (TGFβ) treated cells gave a significantly higher score compared with untreated cells (p < 0.05).

#### Effect of co-culture of AD-MSC with annulus cells on proteoglycan production

**Figure 5:**
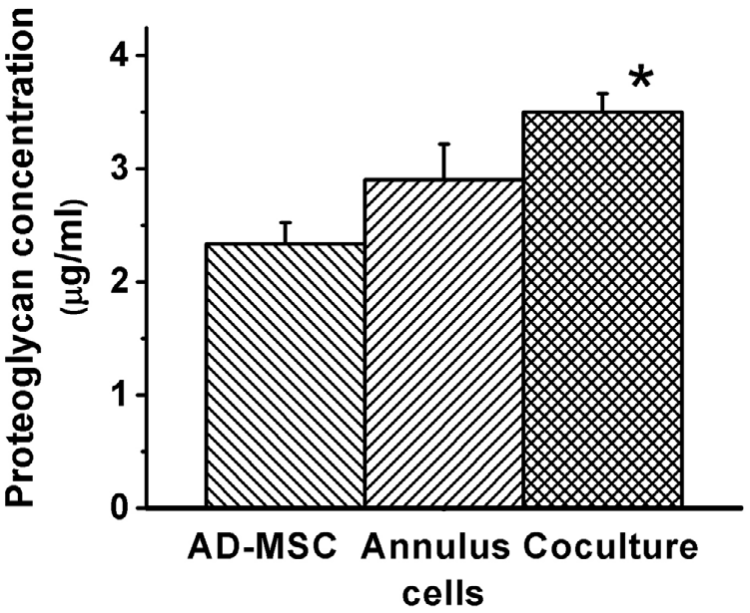
Increase in proteoglycan concentration for 3D co cultured adipose-derived mesenchymal stem cells (AD-MSC) and annulus cells compared with separate culture of AD-MSC and annulus cells alone. Data from eight 1,9-dimethylmethylene blue analyses were examined using repeated measure analysis of variance. p < 0.05.

Evaluation with the DMB assay showed that proteoglycan production increased by approximately 20% (Figure 5). This was assuming a 50:50 ratio of AD-MSC and annulus cells in co-culture compared with the predicted value calculated from the sum of the proteoglycan contents of individual cultures of AD-MSC and annulus cells. Data from eight DMB analyses of 3D cultured AD-MSC alone, annulus cells alone, and AD-MSC and annulus co-cultures were analysed using a repeated measure analysis of variance followed by paired *t*-test analysis. Results were highly significant for AD-MSC compared with co-culture and annulus cells compared with co-culture (Figure 5; p < 0.05). No significant increase in proteoglycan production was seen for disc cells alone treated with CM from AD-MSC. When the ratio of stem cells to disc cells was increased from 1:1, 2:1 or 3:1, proteoglycan production did not change significantly (data not shown). Anti-CFSE antibody labelling clearly showed the presence of labelled disc cells in the 3D culture after two weeks. When disc cells were cultured alone, the CFSE label was clearly visible in almost all cells. Both labelled and unlabelled cells were visible in co-cultures at about a 1:1 ratio, thus verifying that the annulus cells were still present and were not depleted during co-culture.

## Discussion

This study had two main goals: to test whether the stimulation of AD-MSC increased extracellular proteoglycan production and collagen type I using 3D culture in the presence or absence of TGFβ; and to examine the influence of AD-MSC on annulus cells by testing for a synergistic effect on proteoglycan production by 3D co-culture.

AD-MSC were stimulated to produce several known components of the annulus ECM after treatment with TGFβ in 3D culture, confirmed by a 48% increase in proteoglycan content as assayed by DMB analysis and immunohistochemical identification of ECM components. Immunohistochemistry showed that expression of collagen type I, keratin sulphate and decorin was significantly increased in the presence of TGFβ. Chondroitin sulphate and collagen type II showed similar high expression levels in the presence or absence of TGFβ. TGFβ stimulated ECM production is known to occur through SMAD signalling transcription factors and through mitogen activated protein kinase. Chondrogenic gene expression and protein synthesis have been directly correlated with concentration and length of exposure to TGFβ [33]. We speculated that TGFβ stimulation of ECM production by AD-MSC occurred through these pathways. Previously, comparisons of disc and cartilage tissue have identified some ECM similarities. However, intervertebrate disc tissue, in contrast to the articular cartilage phenotype, expresses collagen type I [32]. We show the 3D matrix synthesised by AD-MSC was strongly positive for collagen type I.

TGFβ stimulation of BM-MSC has been previously studied using a micromass pellet culture system. Microarray showed gene expression was found to be closer to annulus fibrosus cells than chondrocytes [32]. Our present work used a 3D collagen sponge for cell growth which, as well as allowing 3D growth and differentiation, also offered a scaffold system to facilitate cell attachment, growth and differentiation. Collagen sponge is flexible with an open porous matrix allowing space for cells to attach and ECM to form. In a surgical situation, it could be sized to fit required dimensions. The matrix will slowly dissolve allowing integration of cells and ECM into the surrounding tissue. Hypoxia and TGFβ have also been used to drive BM-MSC differentiation towards a nucleus pulposus phenotype [34].

Disc cells consist of two distinct cell types, the annulus fibrosus and nucleus pulposus. AD-MSC have feasibility in the repair of both the nucleus pulposus and annulus fibrosus region of the disc. AD-MSC, either in suspension or on an injectable matrix, could be injected directly into the nucleus pulposus where production of proteoglycan and collagen could potentially be stimulated. Before implantation, *in vitro* stimulation with chondrogenic media would be expected to produce ECM richer in collagen type II, the major collagen of the nucleus pulposus. It should be noted that the ECM components identified here are not exclusive to the annulus fibrosis, and are also present in the ECM of the nucleus pulposus and cartilage. There is currently no standard set of genes that 'define' disc cells.

Although disc cells have some chondrocyte-like features, it is important to note that chondrocytes and annulus cells are two completely different mature cell types as illustrated by the matrix they produce and by their biochemistry [12]. Previous work [35] on type II A pro-collagen in the developing human disc found that disc cells show different processing of this pro-collagen than is seen in chondrocytes. Studies by Razaq et al. [36] on the regulation of intracellular pH by bovine disc cells also revealed that the disc cells differ from chondrocytes in that they use a HCO_3_^-^dependent system to regulate intracellular pH. Furthermore, new evidence from our laboratory shows that annulus cells are highly specialised, polarised cells [37].

In the present study we show that co-culture of human annulus and sand rat AD-MSC in 3D culture resulted in a 20% increase in proteoglycan production. Similar to pellet co-culture, AD-MSC and annulus cells were able to coexist and produce a proteoglycan-rich ECM. At present we do not know whether one or both cell types were responsible for the total amount of enhanced synthesis seen. The collagen 3D sponge used here allowed 3D interactions between neighbouring cells, perhaps through contact or growth factor upregulation leading to increased matrix production. TGFβ, IGF-1, epidermal growth factor and platelet-derived growth factor were significantly upregulated in direct cell-to-cell contact co-culture between nucleus pulposus cells and BM-MSC [38]. The synergistic increase in proteoglycan production may be caused by effects such as secreted growth factors released by either cell type enhancing the overall ECM production, or by modification of the microenvironment of the 3D matrix through deposition of ECM components by either the AD-MSC or the annulus cells. Growth factor release *in situ* has been shown to have an effect on mesenchymal stem cells. When the ratio of AD-MSC to annulus cells was increased from 1:1 to 2:1 or 3:1, no further increase or decrease in proteoglycan content was present. A higher ratio of cells may therefore not be required to further stimulate annulus cells.

Previous work from our laboratory has shown the presence of a significant population of senescent cells in the disc, with a greater proportion of senescent cells present in more degenerated discs [3]. Other studies [39,40] also independently verified a high proportion of senescent disc cells. It is possible that senescent disc cells may respond favourably to direct contact with mesenchymal stem cells, potentially allowing resumption of matrix production.

Stimulation of annulus cells by AD-MSC potentially offers a practical approach to autologous disc regeneration and repair. Lu et al. used micromass co-culture to show nucleus pulposus cells could secret soluble factors to direct stem cells towards the nucleus pulposus phenotype [41]. Previous work on interactions of adult mesenchymal stem cells and disc cells by Le Visage et al. [20] showed that annulus, but not nucleus, cells co-cultured in chondrogenic pellets with mesenchymal stem cells had approximately 50% higher proteoglycan content than would be predicted from separate culture alone. In order to test the effect of secreted growth factors, we added CM from AD-MSC cultures to annulus cells in 3D matrix culture. In agreement with a previous study [20] where secreted factors from one cell type were cultured with mesenchymal stem cells, no increase in proteoglycan production was seen.

## Conclusion

Here we investigated growth of AD-MSC and annulus cells in a 3D environment. Adult AD-MSC derived from the sand rat could be stimulated to produce matrix components found in the annulus by exposure to TGFβ in 3D culture. Co-culture of human annulus cells and sand rat AD-MSC in 3D culture resulted in significantly increased proteoglycan production. Results support the hypothesis that AD-MSC may potentially be useful in cell-based therapy for disc degeneration.

## Abbreviations

3D = three dimensional; AD-MSC = adipose-derived mesenchymal stem cells; BM-MSC = bone-marrow derived mesenchymal stem cells; BMP = bone morphogenic protein; CD = cluster of differentiation; CFSE = carboxyfluorescein diacetate succinimidyl ester; CM = conditioned media; DMB = 1,9-dimethylmethylene blue; ECM = extracellular matrix; HBSS = Hank's Buffered Salt Solution; IGF-1 = insulin-like growth factor 1; MSCBM = mesenchymal stem cell basal media; SMAD = small mothers against decapentaplegic homolog; TGFβ = transforming growth factor beta.

## Competing interests

The authors declare that they have no competing interests.

## Authors' contributions

HEG and ENH conceived the study and participated in its design and co-ordination. HT and HEG wrote the manuscript. HT, MK and RD performed all experiments and assays. HT and RD retrieved tissues from animals. JAI performed and modified all immunohistochemical assays. HT and HEG supervised statistical analysis. All authors read and approved the final manuscript.
